# Thalamic volumetric abnormalities in type 1 diabetes mellitus and ‘peripheral’ neuropathy

**DOI:** 10.1038/s41598-022-16699-x

**Published:** 2022-07-29

**Authors:** João L. Novo, José J. Ruas, Leonardo M. Ferreira, Davide Carvalho, Margarida Barbosa, Sofia Brandão, António José de Bastos-Leite

**Affiliations:** 1 Department of Anesthesiology, Hospital de São João, Porto, Portugal; 2 Faculty of Medicine, University of Porto, Alameda do Professor Hernâni Monteiro, 4200-319 Porto, Portugal; 3 Department of Endocrinology, Hospital de São João, Porto, Portugal; 4 Department of Radiology, Hospital de São João, Porto, Portugal

**Keywords:** Magnetic resonance imaging, Biomarkers

## Abstract

We hypothesized that thalamic volumes of patients with type 1 diabetes mellitus (DM) and nonpainful diabetic peripheral neuropathy (DPN) would be reduced relative to thalamic volumes of patients with type 1 DM and painful DPN. We calculated the standardized thalamic volumetric difference between these groups in a pilot sample to obtain a statistical power of 80% at a 5% significance level. Hence, we measured thalamic volumes from 15 patients with nonpainful DPN (10 women, mean age = 49 years, standard deviation [SD] = 11.5) and from 13 patients with painful DPN (8 women, mean age = 43 years, SD = 12.5) by using a manual segmentation approach. A volumetric difference of approximately 15% was found between the nonpainful (mean = 5072 mm^3^, SD = 528.1) and painful (mean = 5976 mm^3^, SD = 643.1) DPN groups (*P* < 0.001). Curiously, a volumetric difference between the left (mean = 5198 mm^3^, SD = 495.0) and the right (mean = 4946 mm^3^, SD = 590.6) thalamus was also found in patients with nonpainful DPN (*P* < 0.01), but not in patients with painful DPN (*P* = 0.97). Patients with nonpainful DPN have lower thalamic volumes than those with painful DPN, especially in the right thalamus.

## Introduction

Diabetes mellitus (DM) is still an incurable disease, for which novel therapeutic approaches are being developed to reduce the incidence of macrovascular and microvascular complications^1^. Diabetic peripheral neuropathy (DPN) is a common microvascular complication of DM, including a wide range of neuropathic involvement associated with significant morbidity^2,3^. The most common category of DPN is the sensorimotor type^3^, of which there are painful and nonpainful subtypes^4^.

Although, by definition, DPN corresponds to a condition involving the peripheral nervous system, magnetic resonance imaging (MRI) studies have demonstrated the occurrence of central nervous system (CNS) abnormalities, namely spinal cord volume loss (detected by measuring the cross-sectional area of the spinal cord)^5^ and gray matter volume loss in somatosensory regions of the brain^6^ (revealed by using voxel-based morphometry [VBM]^7^).

Previous studies have also found metabolic and perfusion abnormalities in the thalamus of patients with type 1 DM and DPN^8,9^. In particular, one of these studies showed lower relative cerebral blood volume and mean transit times in patients with nonpainful DPN than in patients with painful DPN using dynamic susceptibility contrast perfusion-weighted imaging^9^. With respect to thalamic volumetric abnormalities, it was recently established that patients with DPN have thalamic atrophy, irrespective of the concomitance of pain^10^. However, a higher degree of thalamic volume loss was found in patients with nonpainful DPN relative to patients with painful DPN, but such a difference was reported on the basis of a VBM analysis uncorrected for multiple comparisons^6^. It is, however, noteworthy to point out that the thalamus is a very difficult structure to segment using automatic segmentation methods based on signal intensity and contrast of T1-weighted images (T1-WI)^11^, as required for VBM analyses^7^. Therefore, we hypothesized that it could be possible to confirm a reduction in thalamic volumes of patients with type 1 DM and nonpainful DPN relative to those with painful DPN by using a manual segmentation approach^12^.

## Materials and methods

All procedures involving human participants included in this study were in accordance with the ethical standards of the 2013 revision of the Helsinki Declaration and were approved by the local institutional ethics committee (*Comissão de Ética para a Saúde do Hospital de São João*). All patients provided written informed consent to be included.

### Patients

The sample for this study comprised 28 patients with type 1 DM and DPN (18 women, mean age = 46 years, standard deviation [SD] = 12.2). Fifteen patients had nonpainful DPN (10 women, mean age = 49 years, SD = 11.5), and 13 patients had painful DPN (8 women, mean age = 43 years, SD = 12.5).

Patients included for this study fulfilled the American Diabetes Association criteria^13^. Their age was required to be > 18 and ≤ 70 years, and all of them had a duration of disease > 5 years.

To establish the probable occurrence of neuropathic features (e.g., sensation of pins and needles, ants crawling, paresthesia, numbness, cold freezing, allodynia in the lower limbs on a daily basis for at least 3 months, and decreased distal sensation or decreased/absent ankle reflexes), we used the expert panel recommendations published by Tesfaye et al.^14^. We used the Michigan Neuropathy Screening Instrument (MNSI)^15,16^ as a measure of DPN in all patients, which were required to have an MNSI score ≥ 3 to be included. To assess the concomitance of pain, we used the 11-point pain intensity numerical rating scale (PI-NRS)^17^ score. Patients with DPN without chronic pain symptoms (cf., with a PI-NRS score = 0) were classified as having nonpainful DPN. Patients with DPN and pain (i.e., with a PI-NRS score ≥ 1) were required to have a Leeds Assessment of Neuropathic Symptoms and Signs (LANSS) pain scale^18^ score ≥ 12 or a *Douleur Neuropathique* 4 questionnaire (DN4)^19^ score ≥ 4^20,21^ to be classified as having painful DPN.

Exclusion criteria comprised any evidence of CNS or psychiatric conditions, nondiabetic neuropathies, abuse of alcohol consumption, recurrent episodes (> 10) of severe hypoglycemia (i.e., glycemia < 60 mg/dl) during the year prior to baseline assessment, comorbidities precluding MRI scanning, MRI evidence of deep subcortical (lacunar) infarcts and focal thalamic lesions on T2-weighted images (T2-WI), as well as inability to provide written informed consent.

### Magnetic resonance imaging protocol

MRI data were acquired using a scanner operating at 3 Tesla (Magnetom Trio, A Tim System, Siemens, Erlangen, Germany) and equipped with a 12-channel radiofrequency head coil. Axial two-dimensional (2D) inversion recovery (IR) turbo spin echo (TSE) T1-WI were acquired (echo time [TE] = 14 ms, repetition time [TR] = 4300 ms, flip angle = 60°, inversion time [TI] = 400 ms, field of view [FOV] = 240 mm, slice thickness = 2 mm, no interslice gap, number of slices = 44, acquisition matrix = 256 × 256, voxel resolution = 0.9 × 0.9 × 2.0 mm, scanning time = 7:29 min) to segment the thalamus. The choice of parameters (e.g., TI) was based on the optimization of a similar MRI sequence previously used to acquire input images to segment deep gray matter structures^11^.

Single-slab three-dimensional (3D) magnetization-prepared rapid gradient echo (MPRAGE) T1-WI (TE = 3 ms, TR = 2300 ms, flip angle = 9°, TI = 900 ms, FOV = 240 mm, slice thickness = 1.2 mm, number of slices = 160, acquisition matrix = 256 × 256, voxel resolution = 1 × 1 × 1.2 mm, scanning time = 9:14 min) were also acquired to assess measures of brain atrophy. Parameters used to acquire 3D MPRAGE T1-WI were specifically recommended for our scanner, according to the Alzheimer’s Disease Neuroimaging Initiative multicenter study^22^.

### Image analysis

**Figure 1:**
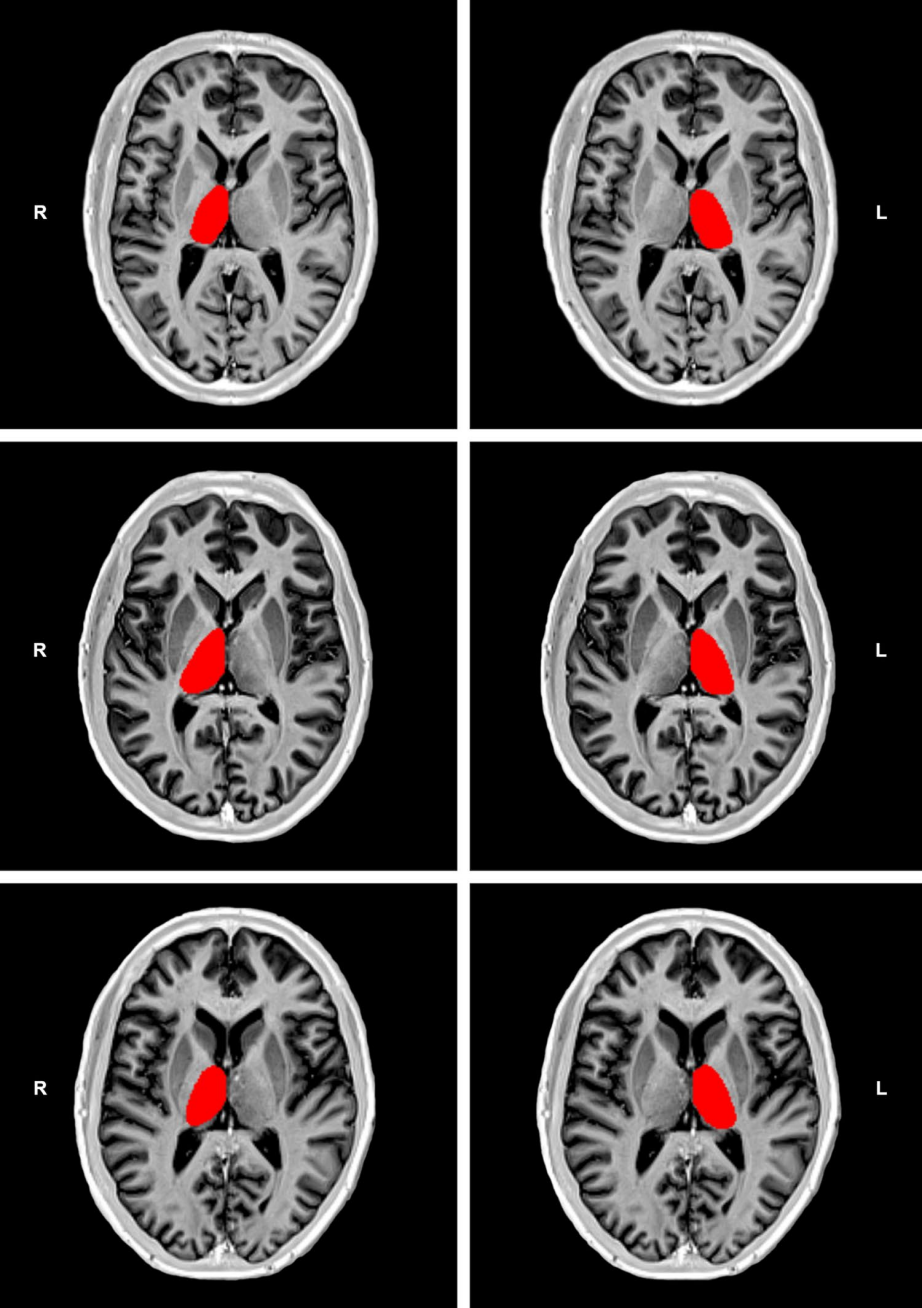
Examples of thalamic segmentation on bias corrected inversion recovery (IR) turbo spin echo (TSE) T1-weighted images (T1-WI) from one patient with nonpainful diabetic peripheral neuropathy (DPN) in the top row and one patient with painful DPN in the middle row. In the bottom row, an example of a similar segmentation procedure on a 46-year-old healthy subject is shown. This example is presented for visualization purposes only, and was randomly selected from a sample of a previous study using similar IR TSE T1-WI to segment deep gray matter structures^11^. Please note the apparent reduction of thalamic volume (especially the thinner cross-sectional thalamic distance) in the patient with nonpainful DPN, especially in the right thalamus, relative to the other examples.

We applied a validated manual segmentation protocol^12^ using the MRIcron software (https://www.nitrc.org/projects/mricron) on bias-corrected 2D IR TSE T1-WI. Although the implementation of a manual segmentation procedure on axial T1-WI implicated a very slight adaptation of the original protocol, it is noteworthy to point out that the reliability analysis of such a protocol has shown that the corresponding intra-class correlation coefficient ranged from 0.95 to 0.98, and the inter-class correlation coefficient ranged from 0.92 to 0.98^12^. Figure 1 shows examples of the thalamic segmentation procedure implemented in the current study. To obtain accurate thalamic volumes, all images including the thalamus were cautiously selected. Care was taken to exclude the metathalamus, epithalamus, subthalamus, hypothalamus, and the choroidal plexuses from measurements. The segmentation procedure was carried out by two observers (A.J. B.-L. and J.J.R.), the more experienced of which (with approximately 2 decades of experience in diagnostic neuroradiology) supervising the other. We also used visual rating scales to assess medial temporal lobe atrophy (MTA)^23^ and global cortical atrophy (GCA)^24^ scores on 3D MPRAGE T1-WI in consensus by two observers (S.B. and A.J. B.-L.). At the time of each assessment, all observers were blinded to the clinical characteristics of the included patients.

### Statistical analysis

Statistical analysis was carried out using IBM SPSS 26.0 (https://www.ibm.com/analytics/spss-statistics-software). Given that the continuous variables analyzed had an approximately normal distribution, we used the Student’s *t*-test to compare their means, namely the independent-samples Student’s *t*-test to compare thalamic volumes at the between-subject level (i.e., between groups), as well as the paired-samples Student’s *t*-test to compare volumes at the within-subject level (i.e., left–right differences). We used the Mann–Whitney U test to compare median scores between groups, the Pearson’s correlation coefficient (*r*) to test correlations between thalamic volumes and age, and the Spearman’s rank correlation coefficient (*r*_*s*_) to test correlations between thalamic volumes, brain atrophy scores, and neuropathy or pain severity scores. Statistical significance was considered when *P *values were < 0.05.

Statistical power estimates were previously carried out on the basis of the Altman’s nomogram^25^ to obtain a statistical power of at least 80% at a 5% significance level and to determine the appropriate total sample size. This was accomplished after calculating the standardized thalamic volumetric difference between the nonpainful and painful DPN groups (i.e., dividing the thalamic volumetric difference between these groups by the SD of the corresponding variable) in a pilot sample including 13 patients with DPN.

### Ethics approval

As stated on the Materials and methods section of this manuscript, “all procedures involving human participants included in this study were in accordance with the ethical standards of the 2013 revision of the Helsinki Declaration and were approved by the local institutional ethics committee (*Comissão de Ética para a Saúde do Hospital de São João*)”.

### Consent to participate

All subjects provided written informed consent to be included.

### Consent for publication

There is consent for publication.

## Results

Table 1 summarizes descriptive results for the most relevant variables in patients with nonpainful and painful DPN. No statistically significant difference was found between groups with respect to age (*P* = 0.19), education (*P* = 0.52), duration of disease (*P* = 0.64), glycated hemoglobin (*P* = 0.77), or body mass index (*P* = 0.99).

**Table 1:** Descriptive results for the most relevant variables in patients with type 1 diabetes mellitus and nonpainful *versus* painful diabetic peripheral neuropathy (DPN), as well as medial temporal lobe atrophy (MTA) and global cortical atrophy (GCA) scores.

	Mean (standard deviation)	
	Non-painful DPN (n = 15)	Painful DPN (n = 13)
Age (years)	49 (11.5)	43 (12.5)
Education (years)	13 (5.5)	10 (3.8)
Duration of disease (years)	34 (15.9)	31 (14.5)
Glycated hemoglobin (%)	8.2 (1.09)	8.0 (1.21)
Body mass index (kg/m^2^)	25.2 (3.79)	25.2 (2.51)
MNSI*^†^	8.8 (3.26)	11.4 (2.51)
PI-NRS^†^	0 (0.0)	5.2 (2.49)
LANSS^†^	N/A	15.4 (5.09)
DN4^†^	N/A	6.2 (2.19)
Left thalamic volume (mm^3^)	5199 (495.0)	5974 (671.7)
Right thalamic volume (mm^3^)	4946 (590.6)	5979 (698.4)
Average left–right thalamic volume (mm^3^)	5072 (528.1)	5976 (643.1)
Average left–right MTA score^†^	1 (0.4)	1 (0.3)
GCA score^†^	1 (0.6)	1 (0.4)

*DN4* *Douleur Neuropathique* 4, *LANSS* Leeds Assessment of Neuropathic Symptoms and Signs, *MNSI* Michigan Neuropathy Screening Instrument, *N/A *not applicable, *PI-NRS* 11-point pain intensity numerical rating scale. *Please note that whereas the Mann–Whitney U test revealed a statistically significant difference of median MNSI scores between groups (*P* < 0.05), no statistically significant difference was found between the corresponding means; ^†^Please note that means and standard deviations of scores are presented to better illustrate the variability of data.

Patients with type 1 DM and DPN had a median and a mean MNSI score of 10 (SD = 3.2). Patients with nonpainful DPN had a median MNSI score of 8 and a mean of 9 (SD = 3.3), whereas patients with painful DPN had both a median and a mean MNSI score of 11 (SD = 2.5). The Mann–Whitney U test revealed a statistically significant difference of median MNSI scores between groups (*P* < 0.05). Patients with painful DPN had a median PI-NRS score of 6, a median LANSS score of 15, and a median DN4 score of 6. The corresponding means were 5 (SD = 2.5), 15 (SD = 5.1) and 6 (SD = 2.2), respectively.

The average left–right thalamic volume in patients with type 1 DM and DPN was 5492 mm^3^ (SD = 734.3). The corresponding volume in patients with nonpainful DPN was 5072 mm^3^ (SD = 528.1) and 5976 mm^3^ (SD = 643.1) in patients with painful DPN. Hence, the nonpainful DPN group had a thalamic volumetric reduction of approximately 15% (i.e., 904 mm^3^) relative to the painful DPN group (*P* < 0.001).

Curiously, a significant volumetric difference between the left (mean = 5198 mm^3^, SD = 495.0) and the right thalamus (mean = 4946 mm^3^, SD = 590.6) was found in patients with nonpainful DPN (*P* < 0.01), which contributed to an almost significant left–right volumetric difference in the entire sample of patients with type 1 DM and DPN (*P* = 0.08). However, no significant difference between the left and the right thalamus was found in patients with painful DPN (*P* = 0.97).

No statistically significant gender-related difference was found with respect to the average left–right thalamic volume in patients with type 1 DM and DPN (*P* = 0.79), either in patients with nonpainful DPN (*P* = 0.37) or in patients with painful DPN (*P* = 0.35).

Significant correlations were found between the left thalamic volume and age (*r* =  − 0.55; *P* < 0.01), the right thalamic volume and age (*r* =  − 0.43; *P* < 0.05), as well as between the average left–right thalamic volume and age (*r* =  − 0.50; *P* < 0.01) of patients with DPN.

No statistically significant correlations were found between thalamic volumes of patients with DPN and MNSI scores, nor between thalamic volumes of patients with painful DPN and PI-NRS, LANSS or DN4 scores. Finally, no statistically significant differences were found between patients with nonpainful or painful DPN with respect to MTA (*P* = 0.89) or GCA (*P* = 0.41), nor significant correlations were found between thalamic volumes and MTA or GCA.

## Discussion

As predicted, our results showed a very significant thalamic volumetric reduction (of approximately 15%) in patients with type 1 DM and nonpainful DPN relative to those with painful DPN. In other words, patients with nonpainful DPN have increased thalamic volume loss relative to patients with painful DPN, as previously anticipated^6^. Curiously, we additionally found a reduction of approximately 5% in the right thalamic volume relative to the left thalamic volume in patients with nonpainful DPN.

As expected, this study confirmed the occurrence of a strong and significant correlation between lower thalamic volumes and increasing age, which corroborates the results of previous studies indicating thalamic volume loss in usual ageing, irrespective of gender^26,27^. Given that no significant difference was found between the mean age of patients with nonpainful DPN and those with painful DPN (i.e., both groups were reasonably matched for age), the possible effect of aging as a confounder seems to be negligible in the current study.

Our study showed that the average left–right thalamic volume was 5492 mm^3^ in patients with type 1 DM and DPN, 5072 mm^3^ in patients with nonpainful DPN, and 5976 mm^3^ in patients with painful DPN. Taking as a reference the results of a previous study reporting thalamic volumes in healthy subjects by using a similar segmentation method^12^, our data suggest that thalamic volumes in patients with type 1 DM and nonpainful DPN are substantially lower than those of healthy subjects (Fig. 1). This is consistent with results of a recently published study^10^. Nevertheless, the corresponding proportion of volume reduction should be confirmed in future studies including healthy control subjects. It is, however, noteworthy to point out that thalamic volumes calculated on the basis of automatic segmentation methods or stereology have always indicated values > 7300 mm^3^ in healthy young adults^28,29^.

The mechanism by means of which the thalamus can be affected in patients with DPN is unknown. Most likely, it can be secondary to loss of sensory input resulting from peripheral nerve damage because strategic parts of the thalamus are key structures in processing sensory stimuli. Alternatively, it has been postulated that a primary involvement of the CNS—leading to neuronal death or dysfunction—might also occur in the so-called diabetic ‘peripheral’ neuropathy. This finding speaks to the aforementioned assumption that thalamic volumes can indeed be reduced in the advanced stage of DPN and is entirely consistent with the reduction of *N*-acetyl aspartate—a marker of neuro-axonal death or dysfunction—found in previous proton magnetic resonance spectroscopy studies^8,10,30^.

According to the neuroanatomical views presented in recent editions of Gray’s Anatomy, “the ventral posterior (VP) nucleus is the principal thalamic relay for the somatosensory pathways. It is thought to consist of two major divisions, the ventral posterolateral (VPl) and the ventral posteromedial (VPm) nuclei. The VPl nucleus receives the medial lemniscal and spinothalamic pathways, and the VPm nucleus receives the trigeminothalamic pathway”. The combination of the aforementioned nuclei is often referred to as the “ventrobasal complex”, which is (along with several other thalamic nuclei) the most relevant target for nociceptive afferents. However, given that small thalamoperforating arteries virtually perfuse all thalamic nuclei, it is plausible that the underlying microvascular complication of DM is widespread and, therefore, not just restricted to the abovementioned nociceptive thalamic nuclei. Nonetheless, in the setting of nonpainful DPN, a selective vulnerability to microvascular pathology of such nuclei could well be demonstrated using a thalamic parcellation method based on diffusion tensor imaging^31^.

The curious finding of an additional degree of volume loss in the right thalamus of patients with nonpainful DPN is consistent with findings of a VBM meta-analysis generally revealing significantly lower right thalamic gray matter density in type 1 DM^32^, as well as with findings indicating that reduced gray matter density in the right thalamus is strongly associated with pain characteristics^33^. Likewise, further right brain hemispheric structures, such as the right insula, the right ventromedial prefrontal cortex, the right nucleus accumbens, and the right amygdala, seem to be involved in the so-called “chronic complex regional pain syndrome^34^”. Accordingly, there is compelling evidence that somatosensory information is preferably processed in the right hemisphere of the brain, especially in some parts of the thalamus^35,36^. In other words, the possible loss of nociceptive neurons in the right thalamus might explain why we found predominant right thalamic atrophy in the nonpainful DPN group.

It is currently uncertain whether thalamic volume loss can be considered a marker of DPN severity. Although it is conceivable to assume that patients with any sort of thalamic dysfunction are at a more advanced stage of DPN, “no biomarker or clear consensus on the clinical definition of either painful or nonpainful diabetic neuropathy exists^4^”. As a matter of fact, we have found both statistically significant lower thalamic volumes and median MNSI scores in patients with type 1 DM and nonpainful DPN relative to patients with painful DPN. Given that the MNSI is considered to be a valid measure of DPN in type 1 DM, the assumption that reduced thalamic volumes can be regarded as a possible marker of DPN severity is at odds with the difference found for the median MNSI score between groups. Further studies are, therefore, warranted to elucidate this apparent jigsaw.

Patients with DM are also at increased risk of developing brain atrophy and cognitive impairment^37^. Nevertheless, there was no evidence of substantial GCA or MTA in our sample^38^.

The fact that we have used a manual segmentation protocol for the thalamus on IR TSE T1-WI probably represents an advantage of this study, given that IR T1-weighted sequences provide images with higher contrast to represent subcortical structures^39^ than other types of T1-WI, such as 3D MPRAGE or spoiled gradient recalled (SPGR) T1-WI. In fact, 3D MPRAGE and SPGR T1-WI are considered to be modalities of choice when detailed anatomical or multiplanar reconstruction is required. Most of the current automatic volumetric methodological approaches rely on their usage to generate input images for brain segmentation^7,40^. Nonetheless, their potential drawback for segmenting the thalamus is a lack of contrast secondary to the higher myelin content in its lateral part—compared to the cortical gray matter and basal ganglia—which diminishes the ability to differentiate the thalamus from the adjacent posterior limb of the internal capsule^11^.

Apart from not including healthy control subjects nor patients without DPN, an additional limitation of the current study is the limited sample size. However, a previous estimation of statistical power was made, which makes it suitable to demonstrate the main difference of interest. Moreover, the exclusion of patients with DPN with lacunar infarcts and focal thalamic lesions on T2-WI—to avoid the concomitance of ischemic small vessel disease as a confounder in terms of image contrast—further contributed to reducing the number of available eligible patients.

Another limitation of this study was its cross-sectional design. In the future, longitudinal studies might demonstrate to what extent thalamic volume loss is a marker of DPN progression and severity. Further multimodal neuroimaging studies, including metabolic and perfusion-weighted MRI sequences (e.g., arterial spin labeling), are also warranted.

In conclusion, our study showed that patients with type 1 DM and nonpainful DPN have lower thalamic volumes than patients with painful DPN, especially on the right side.

## Data Availability

Imaging data are stored in a local repository but can be made available upon request using the e-mail address of the author for correspondence.

## Code availability

The software applied is open source.
